# Minimally Invasive Bilateral Lung Resections and CABG through 5 Ports

**DOI:** 10.1155/2018/9659232

**Published:** 2018-12-13

**Authors:** N. Asemota, M. J. Rouhani, L. Harling, H. Raubenheimer, A. C. De Souza, E. Lim

**Affiliations:** ^1^The University of Nottingham, Queen's Medical Centre, Derby Road, Nottingham NG7 2UH, UK; ^2^Department of Thoracic Surgery, Royal Brompton Hospital, Sydney Street, London SW3 6NP, UK

## Abstract

Minimal access surgery is increasingly popular to reduce postoperative morbidity and enhance recovery. We present a case of a patient who underwent bilateral minimally invasive thoracic and cardiac surgery. An 81-year-old woman was diagnosed with T1aN0M0 left upper lobe small-cell lung cancer and underwent single-port left video-assisted thoracoscopic surgery (VATS) upper lobectomy in 2016. She developed a contralateral right lower lobe nodule and underwent a single-port right VATS wedge resection of the lower lobe nodule, subsequently confirmed as necrotising granulomatous inflammation with acid-fast bacilli, consistent with previous tuberculosis (TB) infection. On postoperative day 1, she had an episode of self-reverting ventricular tachycardia and bradycardia. Subsequent myocardial perfusion scan and coronary angiogram showed significant LV dysfunction and severe coronary artery disease with a left main stem (LMS) lesion. After agreement at MDT, an Endo-ACAB (endoscopic atraumatic coronary artery bypass grafting) was performed, via 3 ports, with the left internal mammary artery anastomosed to left anterior descending artery. She recovered well postoperatively and was discharged. Multiple sequential minimally invasive procedures are now routine and can be performed safely in patients with a complex combination of pathologies. In this case, bilateral single-port (anatomic and nonanatomic) lung resections were undertaken followed by coronary revascularisation with a total of 5 minimal access ports.

## 1. Introduction

The use of minimally invasive surgery has become more widespread over recent years across most surgical fields, likely due to its benefits in reducing length of stay due to faster recovery times as well as causing less surgical trauma. Video-assisted thoracoscopic surgery (VATS) has been firmly established in thoracic surgery, and recent developments have led to increasingly more minimal techniques for cardiac surgery, including, but not limited to, Mini-Mitral and Endo-ACAB (endoscopic atraumatic coronary artery bypass grafting). In this case, we present a rare circumstance of one patient having bilateral minimally invasive cardiac and thoracic surgery sequentially.

## 2. Case Report

An 80-year-old retired woman was referred to our cardiothoracic centre in February 2016 with an incidental finding of 1 cm lung nodule in the left midzone, after having presented to her local hospital with chest discomfort. Her past medical history included previous right nephrectomy for a nonmalignant lesion of the urethra, a previous transient ischaemic attack, polymyalgia rheumatica, hypothyroidism, hypertension, and osteoporosis. Positron emission tomography-computed tomography (PET-CT) showed a T1a N0 M0 left upper lobe cancer with small-cell lung carcinoma confirmed on CT-guided biopsy. The multidisciplinary team decision was for surgical management; therefore, she was admitted for elective lobectomy.

This was undertaken in March 2016 using a single-port VATS technique and included lymph node sampling. Sequential identification, dissection, and division of the pulmonary vessels and bronchi were performed as standard. The procedure was uncomplicated, and the patient was discharged 3 days later.

She then received adjuvant carboplatin/etoposide chemotherapy and was re-referred in December 2016 with a contralateral right lower lobe nodule found on surveillance CT. The patient underwent single-port VATS wedge resection of the nodule in February 2017, which was again uncomplicated. Interestingly, histopathological examination revealed the nodule to be an area of necrotising granulomatous inflammation with acid-fast bacilli, consistent with past tuberculosis, rather than a metastasis.

On postoperative day 1, the patient had a self-resolving episode of ventricular tachycardia following by bradycardia, with chest tightness on minimal exertion. Troponin T was performed which was <20 ng/L. She subsequently underwent a variety of cardiac investigations. Computed tomography coronary angiogram (CTCA) was performed the following day, which showed diffuse disease in all major epicardial vessels, with possible lesions in the left anterior descending (LAD) artery and right coronary artery (RCA), and a coronary calcification score of 1800. A 24-hour Holter investigation revealed bradycardia throughout with rare ventricular ectopics. A subsequent echocardiogram showed normal left ventricular size and function with an ejection fraction of 60–65%, and a myocardial perfusion scan showed an overall ischaemic burden of 7%. Finally, cardiac catheterisation on postoperative day 8 revealed 85% tubular stenosis of the distal left main stem (LMS) artery and minor irregularity in the dominant right coronary artery (RCA).

The patient was symptomatically managed on the ward, and the multidisciplinary team discussed the case. Bearing in mind the patient's comorbidities, a decision was made for Endo-ACAB, rather than conventional cardiac artery bypass grafting (CABG) or percutaneous coronary intervention (PCI), which took place 18 days after the initial thoracic surgery. 3 ports were utilised to harvest the left internal mammary artery (LIMA), and a submammary incision was performed in the 5th intercostal space to enable anastomosis to the LAD. She developed no further complications and was discharged 17 days later. She is awaiting cardiothoracic follow-up.

## 3. Discussion

This is an interesting case of a patient who underwent minimally invasive bilateral lung resections followed by coronary artery bypass grafting, using 5 access ports.

This patient initially had 2 lung resections (ports 1 and 2) via VATS. VATS procedures for lung cancer were first reported in 1991 [1, 2], and since then have become commonplace in thoracic surgery, with increases in use from 2% in 1993 to 14% in 2011 of resection rates in the UK [3], with similar increasing trends across Europe [3]. This is largely due to the increasing evidence of the benefits of VATS for patient care, including reduced pain, reduced hospital stay, and lower risk of postoperative complications [3, 4]. A large trial, such as the VIOLET (video-assisted thoracoscopic lobectomy versus conventional open lobectomy for lung cancer) trial, will enable further scrutiny to establish if there are any long-term clinical benefits of minimally invasive thoracic surgery over open thoracotomies [5].

The patient then underwent coronary revascularisation (ports 3–5), using the endo-ACAB procedure. This procedure, first reported in 1998 [6], involves using endoscopic equipment to harvest the LIMA and anastomose to the LAD, via port access [7]. Despite its early introduction, at a similar time to that of minimally invasive thoracic surgery, the technical difficulties of surgical coronary revascularisation using sole port-access surgery meant an initial shift instead towards the use of thoracotomy incisions, rather than conventional sternotomies [8, 9]. However, there has been a recent trend towards endoscopic cardiac procedures as demonstrated in this case, with evidence of long-term success [10], alongside an increasing prevalence in the use of robotic surgery [7, 8, 11].

In summary, this case highlights the increasing role of minimally invasive techniques in both cardiac and thoracic surgery, which should be considered when managing patients with a number of comorbidities.
